# Pin-tract infection is an important factor associated with pin loosening during external fixation: a prospective analysis of 47 consecutive patients

**DOI:** 10.3389/fcimb.2025.1459205

**Published:** 2025-03-27

**Authors:** Yong-yi Huang, Mou-zhang Huang, Ping Zhang, Chen-sheng Song, Yu Yao, Yu-sheng Yang, Liang-jie Tian, Qing-rong Lin, Ru-hao Han, Hong-wei Xi, Bo-wei Wang, Nan Jiang, Yan-jun Hu

**Affiliations:** ^1^ Division of Orthopaedics & Traumatology, Department of Orthopaedics, Nanfang Hospital, Southern Medical University, Guangzhou, China; ^2^ Guangdong Provincial Key Laboratory of Bone and Cartilage Regenerative Medicine, Southern Medical University Nanfang Hospital, Guangzhou, China; ^3^ Department of Orthopaedics & Traumatology, Yunfu People’s Hospital, Regional Cooperative Hospital of Nanfang Hospital, Southern Medical University, Yunfu, China; ^4^ Department of Trauma Emergency Center, Ganzhou Hospital-Nanfang Hospital, Southern Medical University, Ganzhou, China

**Keywords:** pin loosening, bacterial culture, pin infection, bone quality, insertion angle

## Abstract

**Background:**

The occurrence of pin loosening represents a common issue in the context of external fixation methodologies; nevertheless, a comprehensive investigation into the multifaceted causes of pin loosening, incorporating a multivariate analysis among pin infection, bone quality, and pin insertion angle, is notably absent in current literature. The present study endeavors to pinpoint factors associated with pin loosening through such a multivariate analysis.

**Methodology:**

The study encompassed patients who underwent the removal of external fixators from March 2023 to July 2023. The assessment of pin loosening was executed through the utilization of the pin track score, the pin removal torque value (PRTV), and the radiolucent zone around the pin (RZAP) as depicted in digital radiography (DR) images. Culturing of the pin-bone interfaces was performed, and measurements of the grayscale intensity of cortical bone (GSCB) and pin verticality within DR images were taken. Multivariate analyses were conducted employing a Generalized Linear Mixed-Effects Model (GLMM), Adjusted odds ratios (aORs) with 95% confidence intervals (CIs) were calculated by exponentiating the model coefficients (Exp(β)).

**Results:**

Altogether 47 patients with a total of 220 pins were included for analysis. The mean PRTV was 1.9 ± 2.1 N·m. The correlation analysis between PRTV and RZAP yielded a *P*-value of less than 0.001, signifying a substantial correlation between pin loosening and RZAP. For pins with a PRTV of 0, the RZAP measured 1.9 ± 0.8 mm. The positive rate of bacterial culture was 20%, and the loosening rate was 26.8%. Pin loosening was significantly associated with bacterial infection (aOR = 2.24, 95% CI: 1.03-4.90, *P* = 0.04) and GSCB (aOR = 0.50, 95% CI: 0.38-0.66, *P* < 0.01), but not with pin verticality (aOR = 1.00, 95% CI: 0.93-1.08, *P* = 0.99). Non-HA-coated pins remained significantly associated with bacterial infection (aOR = 8.20, 95% CI: 2.18-30.85, *P* = 0.002), whereas HA-coated pins were not (aOR = 3.44, 95% CI: 0.24-48.76, *P* = 0.36).

**Conclusions:**

Pin loosening was significantly associated with bacterial infection at the pin-bone interface and lower GSCB, but not with pin verticality. Notably, infection strongly predicted loosening in non-HA-coated pins, while HA-coated pins demonstrated higher raw infection rates.

## 1 Introduction

Currently, the management of bone infections, segmental bone defects, complex fractures, and limb deformities poses significant challenges to both patients and orthopedic surgeons. These conditions are characterized by their complex surgical needs and prolonged treatment durations. External fixation serves a crucial role in the treatment of these patient cohorts (Hosny, 2020; Alqahtani et al., 2021). The device is distinguished by its capacity to promote healing without disrupting the injured area, thereby achieving stability by transferring the load from the bone to the pin. As a result, the integrity of the pin-bone interface becomes a crucial factor in maintaining the stability of the external fixation (Moroni et al., 2001). It is now recognized that bone, as a dynamic organ system perpetually undergoing renewal, encounters challenges when it comes into contact with bacteria. Such interaction can induce osteocytes to enter the apoptotic pathway, leading to bone degradation and pin loosening (Wright and Nair, 2010). Therefore, pin loosening has become one of the most frequently occurring complications during such treatment, causing increased patient pain and potential treatment failure. Hence, it is clinically imperative to elucidate the causes of pin loosening to prevent its occurrence and adjust treatment plans accordingly.

Thus far, scholarly inquiry into pins utilized in external fixators has primarily focused on evaluating the robustness of the pin-to-bone interface (Lawes et al., 2004; Roseiro et al., 2014), and on examining the biomechanics of its architecture (Ramlee et al., 2018; Klemeit et al., 2023). Some scholars utilized pin loosening as an observational metric in the examination of infection rates associated with coated and uncoated pins (Bosetti et al., 2002). Additionally, specific research initiatives have employed pin torque values to assess the relationship between pre-treatment pin insertion torque and post-treatment pin removal torque, suggesting that increased pin insertion torque could enhance long-term stability at the pin-bone interface and reduce the occurrence of pin loosening (Lawes et al., 2004). Nevertheless, the above-mentioned investigations predominantly constituted univariate analyses of pin loosening and have not yet explored multi-factor association studies to ascertain whether pin loosening is correlated with multiple critical factors, such as infection, bone quality, and pin insertion angle. Current research into pin tract infection has primarily focused on pin tract nursing, with methodologies primarily centering on pin tract secretions, often overlooking potential discrepancies between infections at the pin-soft tissue interface and pin-bone interface (Jennison et al., 2014). Reliance exclusively on the culture of secretions from the pin-soft tissue interface to assess the pin-bone interface infection is inaccurate, particularly for individuals suffering from bone infections, as a negative bacterial culture at the pin-soft tissue interface does not inherently indicate the absence of infection. The stability of pins is contingent upon the bone, and the quality of the bone is recognized as a critical factor in maintaining stability within internal (Cornell, 2003; Dalstrom et al., 2012) and external fixations (Amin-Al-Tojary et al., 2022). In recent years, studies evaluating bone quality within the frameworks of internal and external fixation have increasingly incorporated systemic osteoporosis into their purview. Nonetheless, there is a notable dearth of research that investigates the effect of local disuse on bone quality and its subsequent impact on the stability of external fixation. It has been established that various configurations of external frames can influence the stability of pins (Klemeit et al., 2023). Nevertheless, despite being the most frequently utilized external fixation framework, it remains uninvestigated whether deviations of pins from the vertical angle are associated with pin loosening.

Upon identifying a knowledge gap, our research endeavored to ascertain factors associated with pin loosening by conducting a multivariable analysis that encompassed pin infection, bone quality, and pin placement angle. Significantly, our objective was to provide a comprehensive foundation for the clinical assessment, prophylaxis, and management of pin loosening.

## 2 Materials and Methods

### 2.1 Study design, inclusion, and exclusion criteria

We executed a prospective cohort study, collecting data from Nanfang Hospital, Southern Medical University. The study concentrated on individuals who had undergone either complete or partial removal of external fixators from March 2023 to July 2023. The criteria for inclusion stipulated the utilization of a unilateral external fixator equipped with a Schanz pin, and the external fixator having been in use for a duration exceeding one month, along with consent to submit pins for examination and review X-rays after pin removal (Bhandari et al., 2005; Dougherty et al., 2006). Exclusion criteria included instances of contamination occurring throughout the pin extraction and culturing process, patients who underwent partial pin removal (such as those undergoing bone transport or lengthening procedures) where the loosening of pins could potentially result in treatment failure and a positive culture result for *Staphylococcus epidermidis*.

### 2.2 Settings and data sources

The investigation encompassed two principal elements: the assessment of pin loosening and the correlation analysis of its causative factors. Pin loosening was evaluated by examining the pin tract reaction, the pin removal torque value (PRTV), and the radiolucent zone around the pin (RZAP) as depicted in digital radiography (DR) images. To augment the objectivity of the assessment, the Checketts-Otterburn scoring system (Checketts AGMaMO, [[NoYear]]) was employed to precisely delineate the status of each individual pin. The PRTV was quantified using a digital torsion wrench throughout the pin extraction process, whereas the RZAP was gauged and computed within the hospital’s Picture Archiving and Communication System (PACS). The factors implicated in pin loosening were distilled to the three most pivotal elements: pin infection, bone quality, and pin placement angle. Pin infection was gauged via bacterial culture at the pin-bone interface, alterations in bone quality were appraised by contrasting cortical bone grayscale values before and after external fixation, and the pin insertion angle was ascertained by evaluating the vertical alignment of the pin and bone at the commencement of treatment. Pin bacterial cultures were executed in the Laboratory Medicine of the hospital, while data on cortical grayscale variations and pin verticality were ascertained within the hospital’s PACS.

### 2.3 Variables and measurements

Variables were classified into two primary categories. The initial category concentrated on evaluating pin looseness, encompassing the pin track score, PRTV, and RZAP within DR images. The subsequent category involved factors associated with pin loosening, including bacterial culture at the pin-bone interface, alterations in the grayscale of the bone cortex within DR images, and the pin’s vertical alignment.

### 2.4 Evaluation of pin loosening

#### 2.4.1 Pin track score

At present, the Checketts-Otterburn grading system is the widely adopted standard for grading external fixator pin tracks, Nevertheless, this criterion regards the external fixation as an aggregate, neglecting to mirror the precise conditions of each pin’s response. To rectify this deficiency, we have refined the scoring methodology to precisely evaluate the clinical manifestations of individual pin trajectories (**Table 1**). The assessment procedure encompassed the observation of erythema, edema, exudation, pain, and the potential for loosening after the removal of the external connecting rod from the external fixator. The torque value indicative of loosening (recorded as 0 N·m) and, where pertinent, the torque measured by a torque wrench in the absence of loosening were also taken into account. Furthermore, the presence of fractures or bone resorption in the vicinity of the pin was evaluated.

**Table 1:** Modified the Checketts-Otterburn grading system.

Grading	Scores	Features
Mild infection	0	No redness, no exudation, no pain or tenderness, and no loosening
	1	There is redness and swelling, and a small amount of exudate
	2	Score 1 + pain or tenderness
Severe infection	3	Score 2 + loose pin
	4	Score 3 + Macroscopic bone resorption around the pin
	5	Score 4 + pathological fracture around the pin track

### 2.5 PRTV measurement

**Figure 1:**
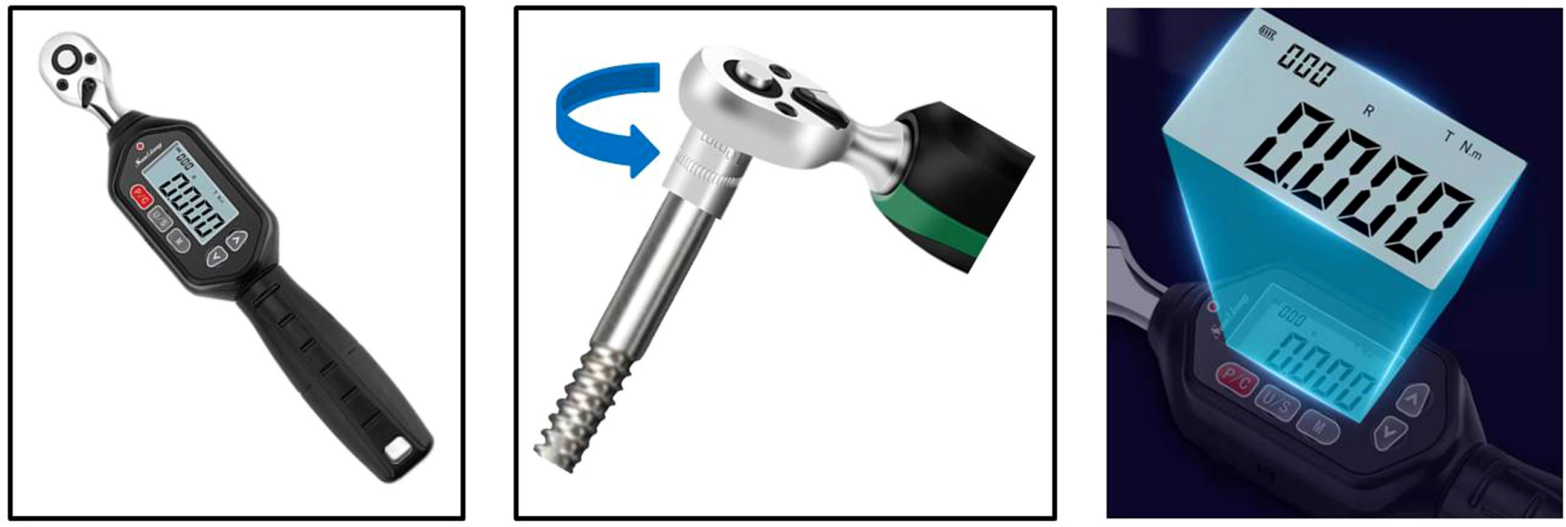
PRTV measurement.

Upon completion of the disinfection process for the pin and pin track, the maximum torque value at the time of pin extraction was determined by employing a digital torque wrench with a precision of 0.001N·m (**Figure 1**). The maximum torque value for each pin was documented before its removal.

### 2.6 RZAP measurement

The RZAP was ascertained by gauging the diameter of the bone tunnel after pin extraction and deducting the diameter of the pin as depicted in the DR images. The DR images before and following pin extraction were concurrently presented on the identical computer screen, and measurements were conducted in the following manner: The tunnel diameter of the cortical region on both sides was measured for the shaft. For the metaphysis, measurements included not only the tunnel diameter of the cortical bone on both sides but also the diameter of the tunnel within the medullary canal. Taking the metaphyseal region as an example, measurements (d_1_, d_2_, d_3_) were obtained at both lateral aspects of the cortex and medullary canal. Three points were measured at each cortex and medullary canal, and the average value was calculated (e.g., d_1_= (d_11_ + d_12_ + d_13_)/3). Subsequently, the diameters of the pin’s solid portion (a_1_, a_2_, a_3_) were measured, ensuring that the measurement locations corresponded with those of the tunnel.

**Figure 2:**
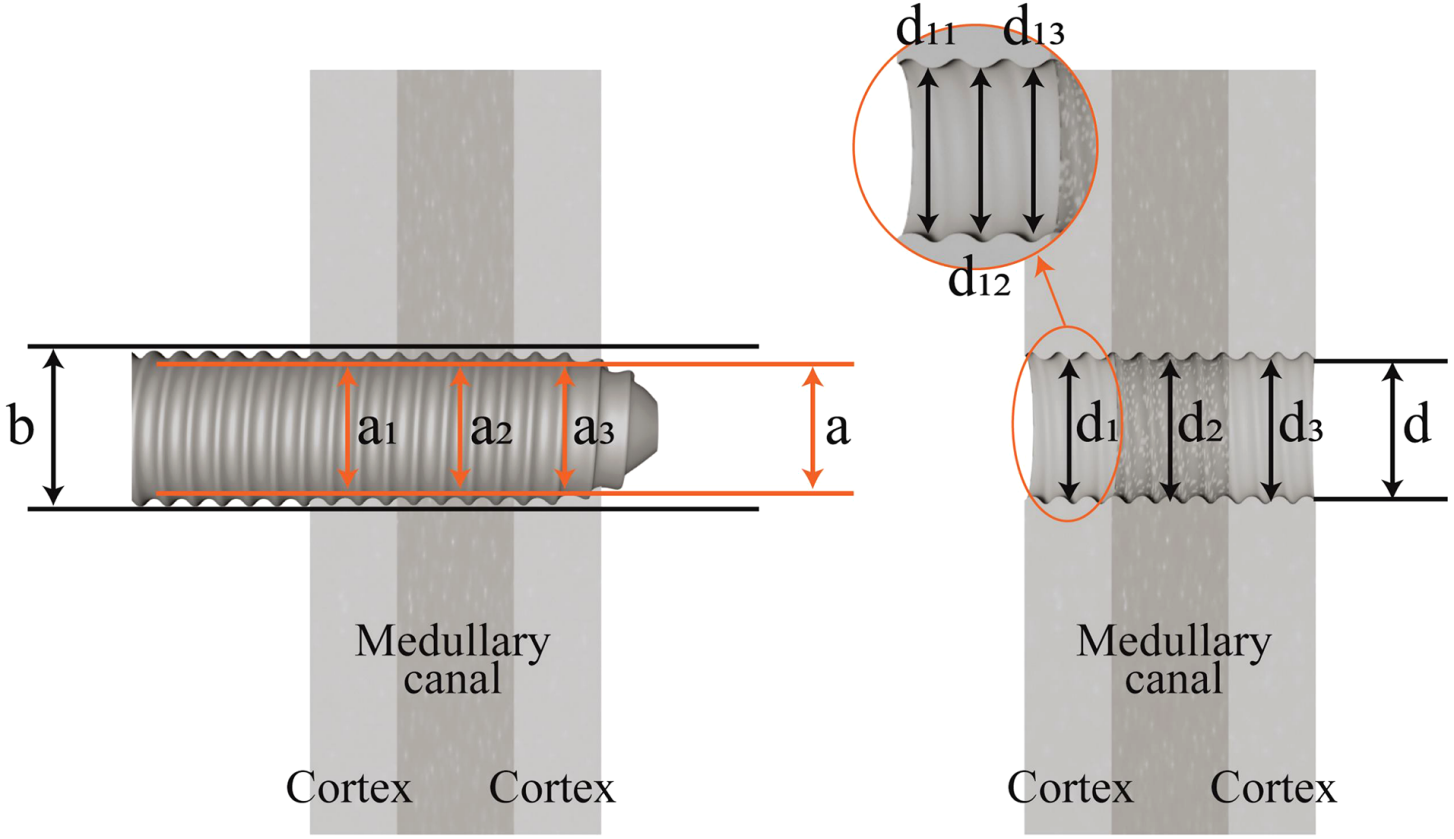
Schematic diagram of bone tunnel diameter measurement after pin removal.

Considering the progressive alteration in thread diameter, maintaining consistency in the measurement location was imperative (**Figure 2**). The variance between these two sets of measurements signified the diameter of the RZAP at each point, specifically denoted as d_1_-a_1_, d_2_-a_2_, d_3_-a_3_. Ultimately, the mean value of the aggregate of these three variances was computed to ascertain the comprehensive diameter of the RZAP (d’). Owing to the difficulties encountered in delineating the boundary of the bone tunnel within the shaft medullary canal, which could introduce substantial inaccuracies, only the diameter of the cortical tunnel on either side of the shaft was assessed. During the measurement procedure, the persistence of the same screen, the application of uniform scale magnification, and the execution of concurrent measurements were employed to mitigate potential errors.

The calculation formula was (metaphyseal): d'=[((d_11_+d_12_+d_13_)/3-a_1_) + ((d_21_+d_22_+d_23_)/3-a_2_) + ((d_31_+d_32_+d_33_)/3-a_3_)]/3.

### 2.7 Identification of bacteria accounting for potential infection of the pin-bone interface

**Figure 3:**
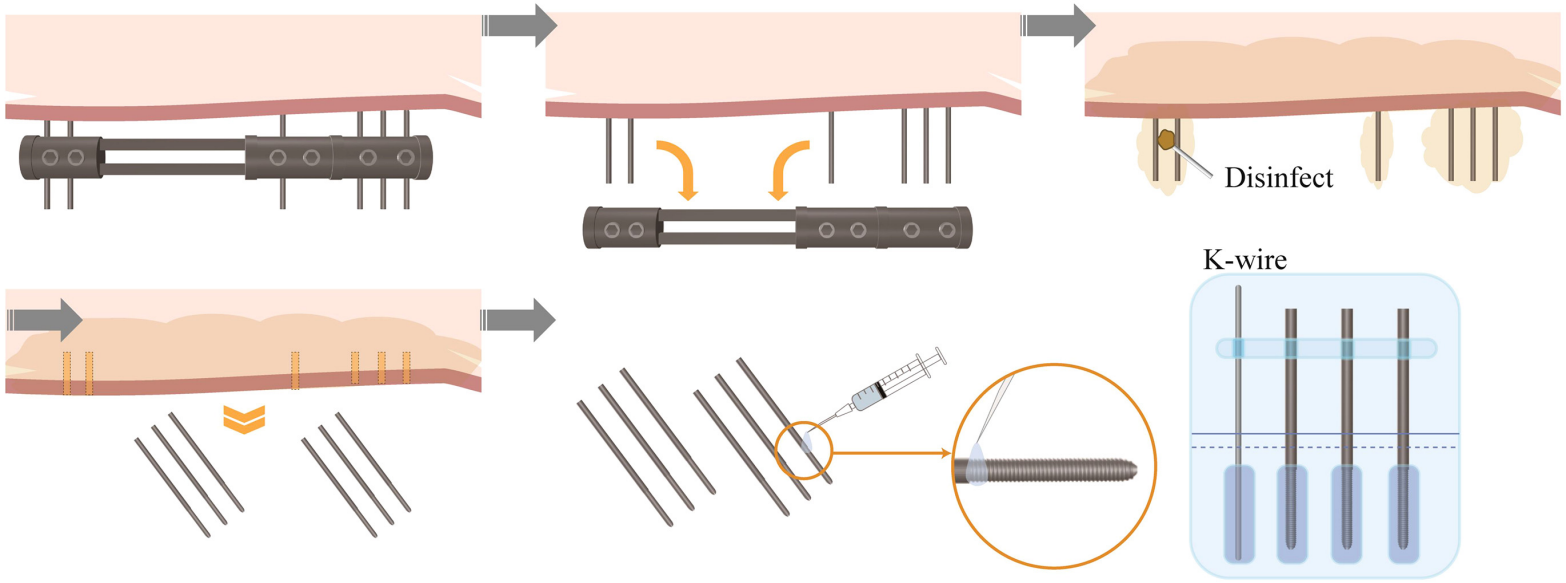
Schematic diagram of the aseptic removal process of the pins.

(1) Pin Removal. The external fixator was entirely or partially extracted within the confines of the operating theatre. Initially, the connecting rod of the external fixator was detached, leaving solely the pins. The pin and its tract were subjected to a meticulous disinfection process using povidone-iodine, and the pin was subsequently extracted employing a torsion wrench, with the PRTV duly noted.
(2) Pin management. After the sterilization of the pins using sterile saline to eradicate blood and visible contaminants, each pin was positioned within a square-type Petri dish, thereby segregating the clean area from the relatively clean area. The extremity of the pin was affixed with a transparent sterile application (**Figure 3**).
(3) Bacterial culture. The bacterial culture process was initiated by securing the Petri dish and dispatching it to the laboratory for analysis. Upon arrival, the pin-bone interface was subjected to trypsin soy agar (TSA) to facilitate bacterial growth. The cultures were maintained in an incubation period spanning 14 days, during which observations were meticulously documented on the first, seventh, and fourteenth days. Specimens yielding positive outcomes were subsequently subjected to additional rounds of bacterial culture and antimicrobial susceptibility testing. At the culmination of the 14 days, the samples were disposed of as medical waste.
(4) Control setting. Throughout the pin configuration phase, a pre-packaged sterile Kirschner wire was positioned within the same Petri dish as a control specimen, ensuring the prevention of cross-contamination. Both the pins and the Kirschner wires were concurrently exposed to TSA for bacterial cultivation. If bacterial presence was identified within the culture of the Kirschner wire, all pins within the Petri dish were classified as contaminated and subsequently excluded from the research.
(5) Precautions. The acquisition of specimens was conducted by the principles of sterility, which encompassed the removal of the external fixator connecting rod, comprehensive disinfection of the pins and the adjacent skin, the rinsing of the excised pins with saline solution to avert the “take-out effect,” and the application of sterile transparent tape for the immobilization of pins and Kirschner wires.

### 2.8 Grayscale changes of cortical bone

#### 2.8.1 Cortical bone grayscale intensity distribution can be used to assess local bone quality

(1) It is widely acknowledged that following the implementation of external fixation, the bone situated between the pins experiences diminished loading due to stress shielding. Bone remodeling, which is affected by the mechanical environment, is hindered by low-loading conditions, which fail to promote osteoblast proliferation. Instead, it enhances osteoblast apoptosis and initiates bone resorption, leading to alterations in cortical bone density (Frost, 1987; Genetos et al., 2005; Giangregorio and McCartney, 2006; Gifre et al., 2015; Gerbaix et al., 2017). This alteration is reflected in a reduction of plate density, accompanied by no substantial decrease in plate thickness (Parfitt et al., 1983). Diagnostic imaging modalities, including X-ray and computed tomography (CT) scans, elucidate disparities in tissue density through the representation of varying grayscale intensities. Dual-energy X-ray absorptiometry (DXA) is extensively utilized for the diagnosis of osteoporosis. Nevertheless, its inherent limitations result in diminished precision when assessing localized disuse osteoporosis in the femur and tibia. CT affords a more precise evaluation of bone trabeculae density (Loundagin and Cooper, 2022), however, its expense and complexity render it inappropriate for routine examination. DR is a frequently employed technique for patient monitoring, utilizing alterations in the grayscale distribution of the bone cortex to evaluate bone quality without necessitating supplementary examinations. Despite significant variations in pixel values within DR images, which can be attributed to differences in projection distance, angle, radiation dosage, and obstructions, the physical attributes of the external fixator pin remain invariant throughout the treatment process. Consequently, its pixel correction value functions as a comparatively stable benchmark, facilitating the assessment of grayscale variations in the same patient across various time intervals.

### 2.9 Measurement methods

**Figure 4:**
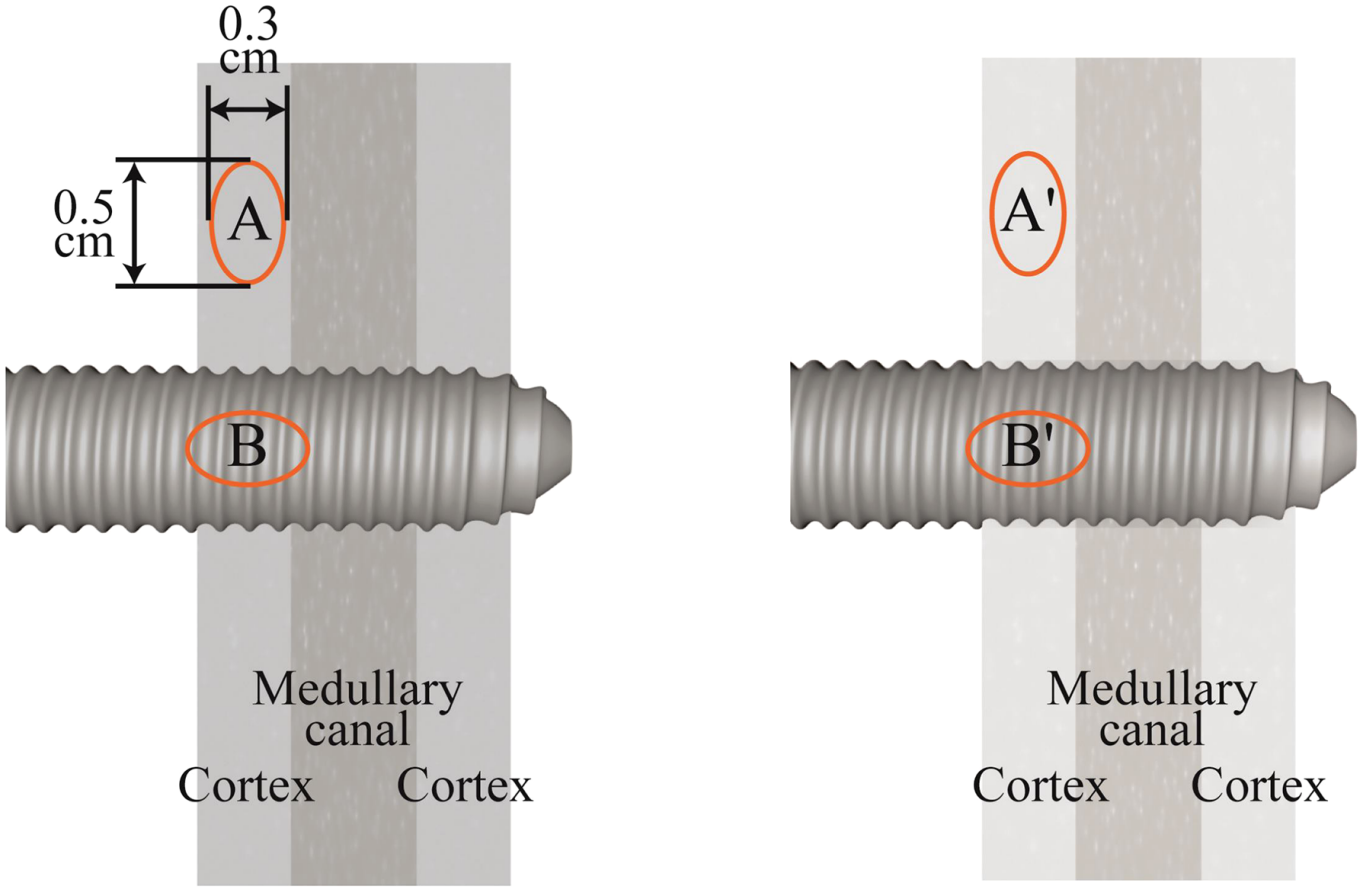
Schematic diagram of grayscale measurement of cortical bone.

The pixel values of the DR images were quantified within the PACS. The region of interest encompassing the cortical area surrounding the pin was delineated as the study zone, with the resultant pixel values designated as A. The pin’s zone, serving as the reference for correction, yielded pixel values represented as B. Subsequently, the pixel correction value, expressed as the ratio A/B, was ascertained. A comparative analysis of the DR outcomes was conducted for each patient, which involved two distinct DR results: one obtained from the initial image taken during the installation of the external fixator and the other from the image captured at the final follow-up appointment. The pixel correction value from the last follow-up (A’/B’) was subtracted from the pixel correction value from the initial installation (A/B), resulting in the alteration of the cortical bone grayscale. This change is denoted by the formula A’/B’ - A/B (**Figure 4**). A positive outcome signifies an enhancement in the grayscale intensity of the cortical bone (GSCB) after the treatment regimen, whereas a negative outcome indicates a diminishment in the GSCB. To minimize measurement error, the measurement function within the PACS post-processing module was employed. During the measurement process, an oval region can be manually delineated to ascertain the average pixel value within this specified area, typically encompassing an ellipse with dimensions of approximately 0.5 cm by 0.3 cm.

### 2.10 Pin verticality

The initial DR image of the patient, secured with the external fixator, was retrieved from the PACS. A straight line was delineated along the center of the pin, and a second straight line was delineated along the center of the shaft, forming an angle. This angle was subsequently measured (∠m), and 90° was deducted from the measured value to ascertain the deviation angle of the pin from the vertical (∠d). The deviation angle (∠d) was utilized to quantify the pin’s verticality, expressed mathematically as ∠d = ∠m-90°.

### 2.11 Statistical analysis

The application of SPSS 30.0 statistical software was executed to conduct statistical analysis. Descriptive statistics were applied to the general data concerning age and the duration of external fixator utilization, which were presented in the form of the mean and standard deviation ( $\bar{\text{S}}$ ± s.d). The outcomes of bacterial culture at the pin-bone interface were classified into negative and positive categories, representing dichotomous variables. In the examination of the association between pin loosening and bacterial culture at the pin-bone interface, as well as GSCB and pin verticality, and the correlation between pin stratification and loosening, PRTV was regarded as a dichotomous variable, with loosening defined as (PRTV < 0.001 N·m) and the absence of loosening as (PRTV ≥ 0.001 N·m). We first conducted bivariate correlation analysis, and after reaching preliminary conclusions, multivariate analyses were conducted employing a Generalized Linear Mixed-Effects Model (GLMM), with a logit link function. The GLMM included patient ID as a random intercept and adjusted for fixed effects of bacterial culture status, GSCB, pin verticality, duration of external fixation, purpose of fixation, gender, HA-coated pin, and non-HA-coated pin. Adjusted odds ratios (aORs) with 95% confidence intervals (CIs) were calculated by exponentiating the model coefficients (Exp(β)). A P-value of less than 0.05 denoted a significant effect. Upon conducting correlation analysis between PRTV and continuous variables RZAP, PRTV was treated as a continuous variable for bivariate correlation analysis. A P-value of less than 0.05 was deemed statistically significant.

## 3 Results

**Figure 5:**
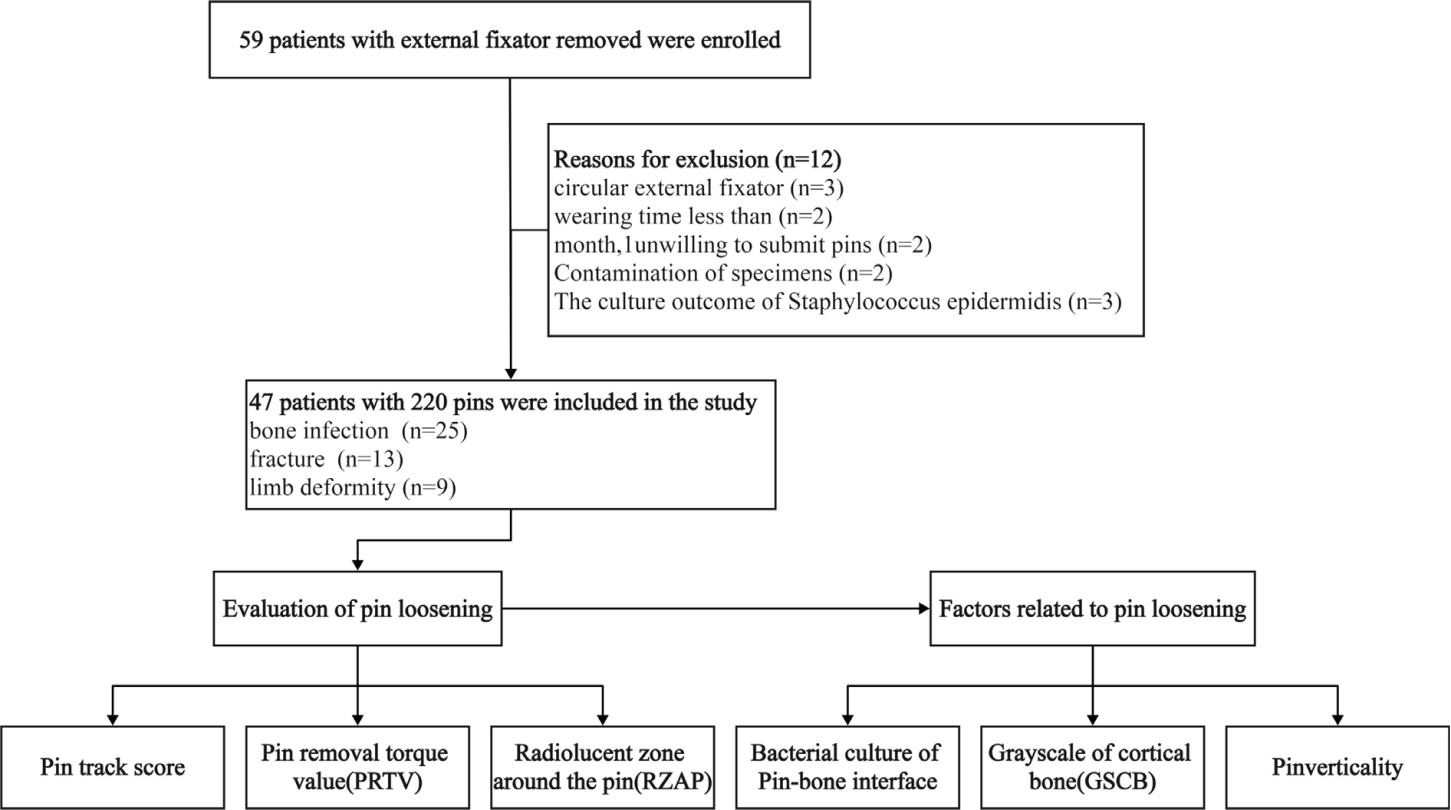
The eligibility selection of the included patients and analysis of potential factors relating to pin loosening.

Initially, a total of 59 patients underwent either complete or partial extraction of the pin of the external fixator. However, 12 patients were excluded due to their failure to satisfy the inclusion criteria. Consequently, the final cohort of the study consisted of 47 patients with a total of 220 pins (**Figure 5**). The demographic profile indicated a male preponderance (32 vs. 15), with a mean age of 43.2 ± 17.7 years. The mean duration of external fixator application was 10.5 ± 7.0 months, with 29 patients having a history of open fractures. The patient cohort encompassed cases of bone infection (25 cases), fractures (13 cases), and limb deformity (9 cases). The external fixator was utilized for single fixation (30 cases), bone transport (12 cases), limb lengthening (3 cases), and limb deformity correction (2 cases). The reasons for the removal of the external fixator encompassed bone healing (30 cases), dynamization (8 cases), and replacement with an internal or external fixator (9 cases).

**Figure 6:**
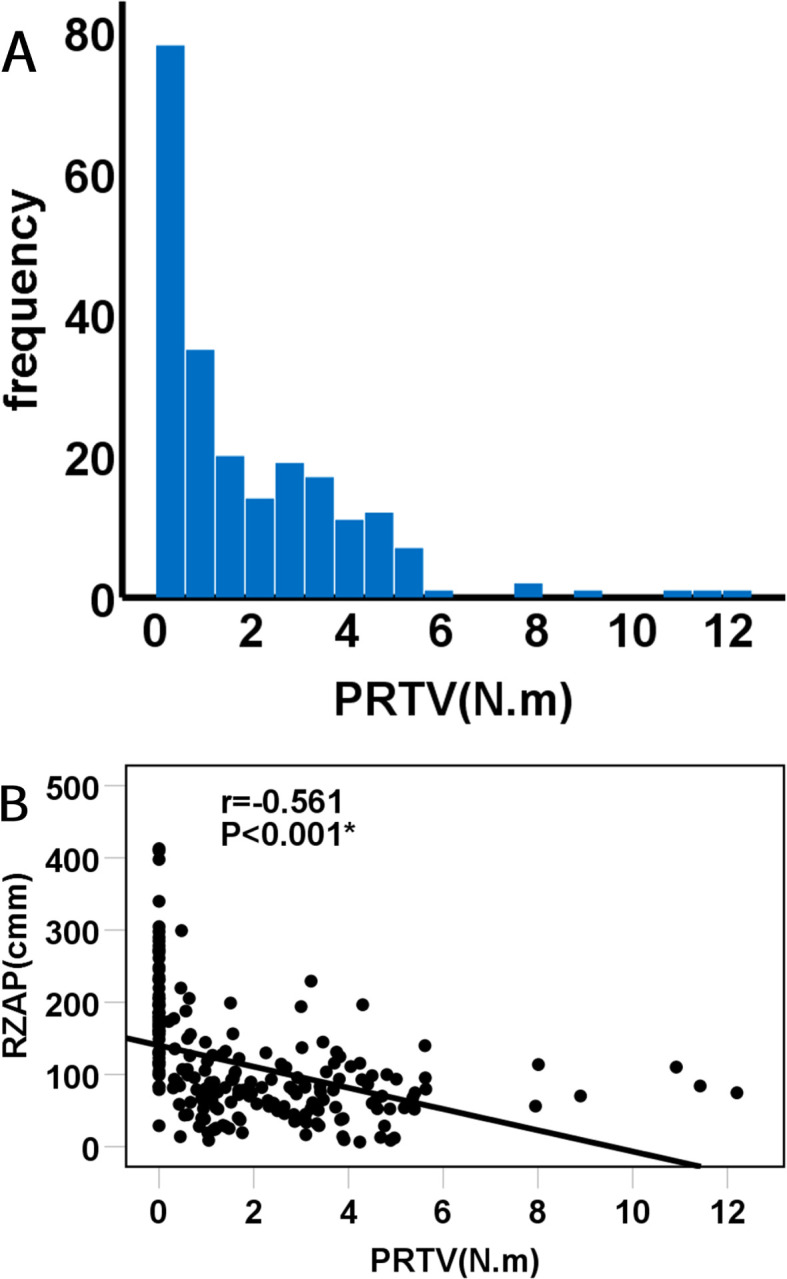
**(A)** histogram visualizes the total pins removal torque value, and **(B)** plot shows a correlation between the PRTV and the RZAP.

Upon further examination of the scores for the 220 pins, it was observed that the predominant number of pins received a score of 2 points (85 pins), succeeded by 0 points (55 pins), 3 points (52 pins), 1 point (27 pins), and a single pin with a score of 4 points. The average PRTV was determined to be 1.9 ± 2.1 N·m, with the lowest PRTV recorded at 0 and the highest at 12.2 N·m (**Figure 6A**). For pins coated with hydroxyapatite (HA), the average PRTV was 3.5 ± 2.3 N·m, in contrast to the non-HA coated pins which exhibited an average PRTV of 0.9 ± 1.1 N·m. A statistical bivariate correlation analysis between PRTV and RZAP revealed a significant negative correlation (*P* < 0.001, r = -0.561), suggesting that increased pin loosening was associated with a higher RZAP (**Figure 6B**). Specifically, when the PRTV was 0, the average RZAP was 1.9 ± 0.8 mm.

Among the 47 patients, it was determined that 17 cases (36.2%) exhibited a positive bacterial culture at the pin-bone interface. Of the 220 pins assessed, 43 (19.6%) demonstrated a positive bacterial culture at the pin-bone interface. It is noteworthy that *Staphylococcus aureus* was the prevalent strain, identified in 31 cases (51.2%, 22/43), whereas 12 cases involved other bacterial species (**Table 2**). In the context of the 25 cases of bone infection, 17 cases tested negative for bacterial culture post-treatment, while 8 cases were positive. Among the positive cases, 2 were consistent with the bacterial types identified during the initial infection, 2 were inconsistent, and 4 could not be compared due to the initial infection bacterial culture being negative. In the 22 cases involving limb deformity and fracture, bacterial culture results indicated 9 positive cases and 13 negative cases (**Table 3**).

**Table 2:** Distributions of the pathogens among the 43 pins with positive culture outcomes.

Pathogen type	Number
*Staphylococcus aureus*	22
*Staphylococcus haemolyticus*	4
*Pseudomonas aeruginosa*	3
*MRSA*	2
*Klebsiella pneumoniae*	2
*Corynebacterium striatum*	2
*Proteus mirabilis*	2
*Escherichia coli*	2
*MRSCN*	1
*ESBLs-KPN*	1
*Staphylococcus caprae*	1
*Staphylococcus lugdunensis*	1

MRSA, Methicillin-resistant Staphylococcus aureus.

MRSCN, Methicillin-resistant coagulase negative staphylococcus aureus.

ESBLs-KPN, Extended Spectrum Beta Lactamases-klebsiella pneumonia.

**Table 3:** Results of bacterial culture at the pin-bone interface (47 cases).

Type	Quantity	Original infection bacteria		Bacterial culture after treatment		Consistency of bacteria before and after treatment	
Bone infection	25	Clear	12	Negative	17		
				Positive	8	Unable to judge	4
		Unclear	13			Consistent	2
						Inconsistent	2
Limb deformities and fractures	22			Negative	13		
				Positive	9		

The incidence of pin loosening was 26.8% (59/220), with HA-coated pins exhibiting a loosening rate of 5.8% (5/86) compared to 40.3% (54/134) in non-HA-coated pins. The overall bacterial infection rate was 20% (43/220), comprising 29.1% (25/86) in HA-coated pins and 13.4% (18/134) in non-HA-coated pins. (**Table 4**). We conducted a bivariate correlation analysis between PRTV of 220 pins and bacterial culture at the pin-bone interface and pin stratification. The results showed that there was no significant correlation between pin loosening and bacterial culture at the pin-bone interface and HA-coated pins (*P* = 0.57 and *P = 0.65*, respectively). However a significant correlation was found between pin loosening of non-HA-coated pins and bacterial culture at the pin-bone interface (*P* = 0.01) After adjusting for the duration of external fixation and the purpose of the external fixation, the multivariable analysis using a Generalized Linear Mixed-Effects Model (GLMM) showed that pin loosening was significantly associated with bacterial infection (aOR = 2.24, 95% CI: 1.03-4.90, *P* = 0.04) and GSCB (aOR = 0.50, 95% CI: 0.38-0.66, *P* < 0.01). Conversely, no significant effect was found between pin loosening and pin verticality (aOR = 1.00, 95% CI: 0.93-1.08, *P* = 0.99) (**Table 5**).

**Table 4:** Number of cases of pin loosening and bacterial culture results at the pin-bone interface.

	Non-HA-coated pin (%)		HA-coated pin (%)		Total (%)	
	Negative	Positive	Negative	Positive	Negative	Positive
No loosening	74 (92.5)	6 (7.5)	57 (70.4)	24 (29.6)	131 (81.4)	30 (18.6)
Loosening	42 (77.8)	12 (22.2)	4 (80)	1 (20)	46 (78.0)	13 (22.0)
Total	116	18	61	25	177	43

**Table 5:** Results of the Generalized Linear Mixed-Effects Model (GLMM) for pin loosening risk: Fixed effects of bacterial culture, GSCB, and pin verticality.

Model Term	Coefficient	P-value	aOR	95%CI (aOR)	
				Lower	Upper
Positive Culture	0.808	0.043	2.243	1.026	4.902
GSCB	-0.697	<0.001	0.498	0.378	0.657
verticality	-0.001	0.987	0.999	0.925	1.079

The model was adjusted for the duration of external fixation and the purpose of the external fixation. aOR, Adjusted Odds Ratios, calculated as Exp (Coefficient); GSCB, grayscale intensity of cortical bone.

After stratifying the analysis into non-HA-coated pins (n = 134) and HA-coated pins (n = 86), a generalized linear mixed-effects model (GLMM) adjusted for gender and duration of external fixation revealed distinct associations with bacterial infection. Non-HA-coated pins were significantly associated with bacterial infection (aOR = 8.20, 95% CI: 2.18-30.85, *P* = 0.002), whereas HA-coated pins showed no statistically significant association (aOR = 3.44, 95% CI: 0.24-48.76, *P* = 0.36). Compared with non-HA-coated pins, HA-coated pins were a protective factor against pin loosening (aOR = 0.03, 95% CI: 0.01-0.21, *P* < 0.01) (**Table 6**).

**Table 6:** Stratified GLMM analysis by coating type: Association between pin coating type and pin loosening.

Model Term	Coefficient	P-value	aOR	95%CI (aOR)	
				Lower	Upper
HA-coated pin	-3.441	<0.001	0.032	0.005	0.209
Positive HA-coated pin	1.236	0.359	3.442	0.243	48.756
Positive non-HA-coated pin	2.104	0.002	8.195	2.177	30.849

The model was adjusted for gender and the duration of external fixation. aOR: Adjusted Odds Ratios, calculated as Exp(Coefficient); HA, Hydroxyapatite; “Positive” indicates bacterial culture positivity at the pin-bone interface.

## 4 Discussion

In the present study, our objective was to assess pin looseness, commencing with the modification of the Checkets-Otterburn pin track grading methodology (Checketts AGMaMO, [[NoYear]]). The modification was conceived to better correspond with the assessment of individual pin tracks within our study, enhancing efficiency and user-friendliness for medical professionals and patients alike. The quantified torque value functioned as a precise metric for evaluating pin looseness, serving as both a binary and continuous variable for the analysis of associations with bacterial culture at the pin-bone interface, RZAP, alterations in GSCB, and pin insertion perpendicularity. This study substantiated a correlation between pin loosening and RZAP, demonstrating a significant association when the pin was loose (PRTV was 0), resulting in a mean RZAP of 1.94 mm. This outcome was consistent with the study conducted by Pettine et al., wherein an RZAP of 1 mm or greater indicated severe pin loosening (with a 96% incidence of pin loosening). The reduction of RZAP could potentially be accomplished through the enhancement of the radial preload of pin (Hyldahl et al., 1991), offering clinical insights for pin loosening assessment and prevention.

Reports in the literature suggest a broad spectrum of infection incidences associated with pin tracks of external fixators, ranging from 3% to 80% (Collinge et al., 1994; Parameswaran et al., 2003). In our cohort, bacterial culture analysis at the pin-bone interface revealed an infection incidence of 19.6% (43/220), which included twelve distinct bacterial pathogens. *Staphylococcus aureus* was identified as the predominant species, constituting 51.2% of the identified infections (Harris and Richards, 2006; de Breij et al., 2016; Slate et al., 2018). It was noteworthy that the positive rate for the bacterial culture of HA-coated pins surpassed that of non-HA-coated pins, reaching 29.1% (25/86) in contrast to 13.4% (18/134). This finding agreed with the outcomes reported by Pommer et al (Pommer et al., 2002), who had reported that the infection rates of 30% for coated pins and 21% for non-coated pins. Nonetheless, Stoffel et al (Stoffel et al., 2023). noted similar infection incidences between HA-coated and non-HA-coated pins (45.7% vs. 48.5%). Despite the elevated removal torque value for HA-coated pins (3.52 N·m in contrast to 0.88 N·m), HA-coating does not possess antibacterial properties, and it did not decrease the infection rate of pins.

In adjusted analyses, bacterial infection significantly increased loosening risk overall (aOR = 2.24, *P* = 0.04). Stratification by coating type showed this effect was driven by non-HA-coated pins, where infection markedly elevated loosening risk (aOR = 8.20, *P* = 0.002). The seemingly paradoxical combination of a low bacterial infection rate (13.4%) and a high adjusted odds ratio (aOR = 8.20) in non-HA-coated pins can be explained by two factors. First, odds ratios in logistic regression models tend to overestimate relative risk when the outcome (pin loosening) is not rare, as demonstrated in our cohort where 40.3% of non-HA-coated pins loosened. Second, even a low incidence of infection may critically destabilize the pin-bone interface through biofilm-induced osteolysis, particularly in the absence of HA coating’s osseointegration protection. This “all-or-none” effect is consistent with Pommer et al.’s prior study that reported a disproportionately high aOR of 5.2 for loosening in the low-infection subgroup (Pommer et al., 2002). In contrast, HA-coated pins showed no significant association between infection and loosening (aOR = 3.44, *P* = 0.36), likely stemming from their microporous surface structure, which promotes bone ingrowth but also provides niches for bacterial adhesion. The hydroxyapatite (HA) coating may enhance bone-implant bonding strength, as evidenced by the significantly reduced loosening risk in HA-coated pins compared to non-HA-coated pins (aOR = 0.03, *P* < 0.01). This protective effect persisted even in the presence of infection, suggesting that HA coatings mitigate infection-related mechanical destabilization (Goodman et al., 2013; Stoffel et al., 2021).

In conclusion, HA-coated pins demonstrate a dual clinical profile: they significantly reduce loosening risk (aOR = 0.03) compared to non-HA-coated pins, likely due to improved osseointegration and higher initial torque values. However, the porous HA coating may paradoxically facilitate bacterial colonization, as suggested by the higher infection rate in HA-coated pins (29.1%) than in non-HA-coated pins (13.4%). This dual trade-off (enhanced bone bonding versus elevated infection rates), highlights the urgency to develop next-generation coatings that synergize osteoconduction (via HA or similar materials) with localized antibacterial strategies (e.g., silver nanoparticles, antibiotic-eluting polymers). Such hybrid coatings could mitigate infection risks without compromising mechanical stability, addressing the limitations observed in current HA-coated pins.

The extant literature lacks conclusive evidence regarding the interpretation of positive bacterial cultures at the pin-bone interface, specifically whether they indicate the emergence of new infections or the persistence of the original infection. The current study aims to clarify this issue. Among the 25 patients diagnosed with bone infections, 17 had negative cultures, while 8 tested positive following treatment with an external fixator. In the instances where cultures were positive, only 2 matched the bacterial strains identified before the treatment, suggesting that these instances were most likely due to continuations of the initial infection. The remaining 6 cases exhibited different bacterial strains. Determining whether these 6 cases represent new infections is challenging, especially given that 4 initially had negative bacterial culture results. This ambiguity may be due to the inherently low positive rate of bacterial cultures, necessitating the use of supplementary methods such as bacterial gene sequencing, tissue culture, or internal plant culture to improve detection rates (Raskin et al., 2006; Chen et al., 2022; Jiang et al., 2022). An additional factor that may contribute to the difficulty in diagnosing osteomyelitis is the administration of antibiotics before the collection of cultures or the premature discontinuation of such medication. Hence, whether it is caused by exogenous or endogenous infection is still unclear, and more studies are needed to confirm. Despite these challenges, the pin-bone interface culture remains a crucial instrument for determining the eradication of infection and the detection of any subsequent infections. Furthermore, our research revealed that among 22 patients who initially presented with limb deformities and fractures without infection, 9 cases demonstrated positive bacterial cultures at the pin-bone interface following treatment with an external fixator. This finding underscores the potential for external fixator pins to induce new infections, a phenomenon that is closely linked to the communication dynamics of the pin track with the external environment (Jennison et al., 2014).

Upon examination of the bacterial culture results at the pin-bone interface, the following recommendations are proposed: In scenarios where an external fixator is utilized as the definitive method of fixation, and the bone has mended with a negative bacterial culture at the pin-bone interface, no specific intervention is necessary for the removal of the external fixator. For individuals exhibiting a positive bacterial culture, it is advised to administer oral antibiotics that are sensitive to the identified bacteria, until symptoms such as redness, swelling, exudation, or pain at the pin tract have subsided. Patients with a pre-existing bone infection should be subjected to routine X-ray evaluations. The staged fixation technique necessitates the employment of an external fixator. In instances where the substitution of the internal fixator is deemed requisite, and the bacterial culture at the pin-bone interface returns negative outcomes, it is advised that the surgical intervention be postponed for a duration of 5 to 7 days until the absence of redness, swelling, and exudation within the pin tract is observed. In the occurrence of a positive bacterial culture, the subsequent treatments are advised: the replacement of a new external fixator; implementation of internal fixation while ensuring to circumvent the infected pin tract (Potter et al., 2019), and sensitive antibiotics should be administered post-operation, acknowledging the persisting risk of internal fixation infection. Upon completion of antibiotic therapy, internal fixation should be pursued after the normalization of inflammatory indices. In instances where bone infection is present, it is advisable to conduct re-debridement and subsequently fill with calcium sulfate bone powder, followed by internal fixation in the secondary stage (Pairon et al., 2015).

The grayscale intensity of cortical bone (GSCB) serves as a quantitative indicator of local bone quality. In our adjusted analysis, higher GSCB values were associated with a 50% reduction in pin loosening risk (aOR = 0.50, 95% CI: 0.38–0.66, *P* < 0.001). This finding aligns with the biomechanical principle that denser cortical bone (reflected by elevated GSCB) enhances pin-bone interface stability, thereby resisting micromotion and loosening. Nevertheless, clinical observations suggest variability in bone adaptation under external fixation. Prolonged immobilization without weight-bearing may lead to disuse osteoporosis, as evidenced by Smith et al (Smith et al., 1993). who reported a 70% incidence of acute local osteoporosis in tibial fracture patients treated with external fixation. Importantly, our results imply that early weight-bearing protocols could synergistically improve bone quality (increasing GSCB) and reduce loosening risk. Enhanced mechanical loading may stimulate bone remodeling, counteracting osteodystrophy while optimizing fixation stability. Thus, we strongly advocate for the timely initiation of controlled weight-bearing in patients with external fixators, as it may dualistically mitigate post-traumatic osteoporosis and mechanically reinforce the pin-bone interface through GSCB augmentation (Tandon et al., 1995).

In our study, structural external fixators were utilized for fundamental stabilization, and pins positioned within the vertical diaphysis demonstrated robust biomechanical stability (Biliouris et al., 1989; Harari, 1992; Aro et al., 1993). Upon insertion at an angle (Chao and Hein, 1988), the cantilever loading exerted on the pin at the pin-bone interface (particularly when the patient is required to bear weight post-surgery) can generate stress that surpasses the yield strength of the cortical bone, potentially resulting in bone resorption and loosening (Giotakis and Narayan, 2007). Nevertheless, our study found no significant association between pin verticality and loosening risk (aOR = 1.00, 95% CI: 0.93–1.08, *P* = 0.987). The mean pin deviation angle in our cohort was minimal (3.41°), likely due to rigorous intraoperative fluoroscopic guidance. These results suggest that minor angular deviations within a controlled surgical protocol do not measurably affect stability. Nevertheless, adherence to standardized pin placement remains critical, as larger deviations in less optimized settings may still induce stress concentrations. Additionally, increasing preloading force during pin insertion could further mitigate loosening risks (Klemeit et al., 2023).

The study’s strengths encompass the quantification of pin loosening, the evaluation of pin loosening via the RZAP, the precise assessment of pin infection through bacterial culture at the pin-bone interface, and the multivariate analysis of pin loosening. However, it is imperative to recognize certain limitations. Initially, the profound location of the bacterial culture at the pin-bone interface poses significant challenges in ensuring sterile conditions during the extraction process. This is especially true for pins situated in the femur, which have lengthy channels of soft tissue that are prone to contamination from skin and soft tissue secretions during the removal procedure. Despite adopting measures such as aseptic surgical techniques, pin flushing, and partitioned culture methods, the complete elimination of the “bring out effect” remains a formidable challenge. Secondly, the study fails to incorporate dynamic factors that influence pin loosening, including the loading weight and the frequency of patient activity post-discharge, which are difficult to objectively quantify (Harari, 1992; Pommer et al., 1998). Thirdly, factors such as intraoperative thermal injury, pin insertion torque, and pin oxidation (Seitz et al., 1991; Manoogian et al., 2017), which might influence pin loosening, are not encompassed within the scope of this study and merit examination in subsequent research endeavors. Finally, the limited sample size in this study underscores the necessity for an expanded sample size to achieve more robust findings in future studies.

## 5 Conclusions

In summary, the assessment of pin loosening can be directly conducted through the utilization of pin track score and PRTV, with RZAP serving as a foundation for indirect evaluation. Pin loosening was significantly associated with bacterial infection at the pin-bone interface and lower GSCB, but not with pin verticality. The null association with verticality may reflect standardized surgical protocols minimizing angular deviations. Despite their higher raw infection rates, HA-coated pins significantly reduced loosening risk compared to non-HA-coated pins. While infection strongly predicted loosening in non-HA-coated pins, this association was attenuated in HA-coated pins. These findings support routine bacterial culture to guide early fixation revision in infected non-HA pins and highlight HA coatings as a viable strategy to mitigate loosening, pending solutions to their infection propensity.

## Data Availability

The raw data supporting the conclusions of this article will be made available by the authors, without undue reservation.
